# Effects of cigarette smoking on the growth of *Streptococcus mutans* biofilms: An *in vitro* study

**DOI:** 10.1371/journal.pone.0259895

**Published:** 2021-11-15

**Authors:** Ye Han

**Affiliations:** Department of Preventive Dentistry, School of Dentistry, Institute of Oral Bioscience and BK 21 Plus Program, Jeonbuk National University, Jeonju, Republic of Korea; Sejong University, REPUBLIC OF KOREA

## Abstract

The increased incidence of dental caries by cigarette smoking (CS) has been widely reported in epidemiological studies, but the relationship between CS and cariogenic biofilm growth has been rarely studied. This study aims to investigate the effects of CS exposure on the growth and virulence of *Streptococcus mutans* biofilms (*S*. *mutans*). Briefly, *S*. *mutans* biofilms were formed on saliva-coated hydroxyapatite disks, which were exposed to CS 1, 3, and 6 times per day, respectively. In addition, *S*. *mutans* biofilms without CS exposure were considered as the control group. Acidogenicity, dry weight, colony-forming units (CFUs), water-soluble/insoluble extracellular polysaccharides (EPSs), and intracellular polysaccharides (IPSs) were analyzed and confocal laser scanning microscopy (CLSM) images of 74-h-old *S*. *mutans* biofilms were obtained. The lowest accumulation of biofilms and EPSs were detected in the 6 times/day CS exposure group compared with those of the control group and other CS exposure groups in 74-h-old *S*. *mutans* biofilms. CLSM also revealed the lowest bacterial count (live and dead cells) and EPSs biovolume in the six times/day CS exposure group in 74-h-old *S*. *mutans* biofilms. CS exposure inhibited the growth of *S*. *mutans* biofilm *in vitro* study, the anti-cariogenic biofilm formation was enhanced with a dose (frequency)-dependent at which frequency has more influence in the present findings.

## Introduction

Dental caries is a dental biofilm-related disease [1]. An important contributing factor in the development of dental caries is the change of microbial properties in dental biofilm. The increase of aciduric and acidogenic bacteria in the microorganism is of great significance in the pathogenesis of dental caries [1]. Through glycolysis, acidogenic bacteria reduce pH in the dental biofilm developed on the surface of teeth that are exposed to dietary sugar [1]. The low pH environment further accelerates the growth of acidity and acidogenic bacteria including *mutans streptococci*, other *acidogenic streptococci*, *lactobacilli*, and *bifidobacteria* [2]. Although the composition of cariogenic biofilm microflora is complex [3], *S*. *mutans* is considered one of the important etiological agents for the development of dental caries [4, 5]. *S*. *mutans* can synthesize EPSs produce glucosyltransferases with sucrose as a substrate. EPSs contribute to bacterial adhesion on the tooth surface, colonization, and formation of plaque biofilm, which are important cariogenic factors of *S*. *mutans*. Besides, acid production through carbohydrates and acid tolerance in low pH is also the major cariogenic factors of *S*. *mutans* that result in the loss of local hard tissue and the initiation of the cariogenic process [6].

CS is an important factor affecting the progress of dental caries. Numerous epidemiological studies around the world have reported a close relationship between smoking and the occurrence of dental caries [7–11]. An animal study reported that CS exposure expands the caries-affected area in the maxillary molars of the rat [12]. This is further confirmed by evidence from epidemiological studies associating CS with high caries prevalence [13].

To the best of the author’s knowledge, the relationship between CS and the growth of cariogenic biofilm (*S*. *mutans* biofilms) has been rarely analyzed, which is of great significance to study the influence of CS on the growth of *S*. *mutans* biofilms and dynamic bacterial equilibrium for further understanding the relationship between smoking and high caries prevalence. This study aimed to investigate the influence of CS on the growth, virulence (EPSs and acidogenicity), and viability of cariogenic biofilms *in vitro* by using the *S*. *mutans* biofilm model.

## Materials and methods

### *S*. *mutans* biofilms formation and CS experimental scheme

**Fig 1:**
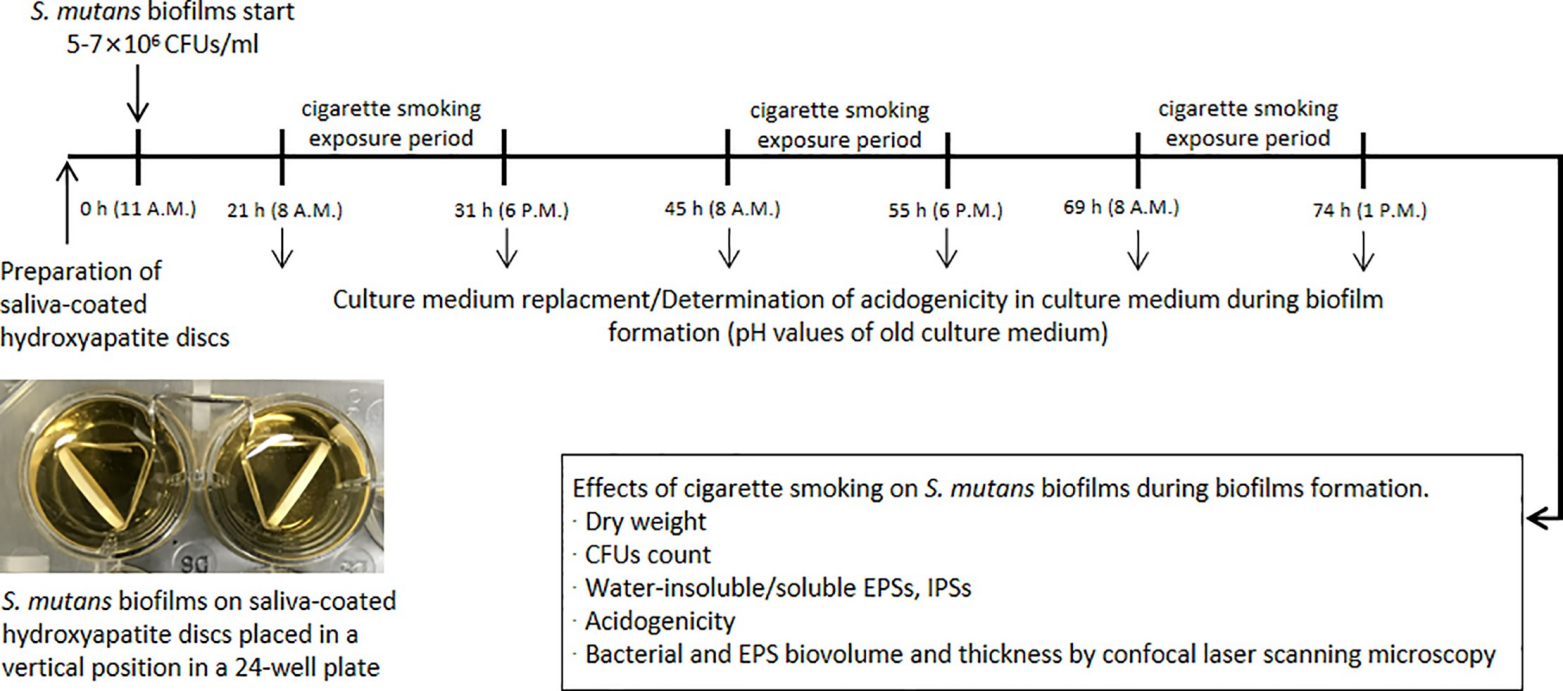
*S*. *mutans* biofilms formation and experimental scheme.

*S*. *mutans* UA159 (ATCC 700610; serotype c) biofilms were formed on saliva-coated hydroxyapatite discs (2.93 cm^2^; Clarkson Chromatography Products, Inc., South Williamsport, PA, USA) placed in a vertical position in 24-well plates. Briefly, an adult male (non-smoker) was selected for oral saliva collection. Hydroxyapatite discs were incubated in filter-sterilized (0.22-μm low protein-binding filter) saliva (3 ml/disc) for 1 h at 37°C. For biofilms formation, the saliva-coated hydroxyapatite discs were transferred to a 24-well plate containing brain heart infusion (BHI; D-ifco, Detroit, MI, USA) broth with 1% (w/v) sucrose and *S*. *mutans* (5–7×10^6^ colony-forming unit (CFU)/ml) (3 ml/disc). The biofilms were grown at 37°C with 5% CO_2_ until 74 h (Fig 1). After 21 h (initial biofilms growth), *S*. *mutans* biofilms were divided into 4 groups. Experiment group 1 (control group) did not receive CS exposure and are exposed to the air six times per day (at 8, 10, 12 a.m., 2, 4, 6 p.m.). Experiment group 2 was exposed one time per day (at 10 a.m.) with CS and five times per day (at 8, 12 a.m., 2, 4, 6 p.m.) with air, a total of three times CS exposure in 74-h-old biofilms. Experiment group 3 was exposed three times per day (at 10 a.m., 2, 6 p.m.) with CS and three times per day (at 8, 12 a.m., 4 p.m.) with air, a total of seven times with CS exposure in 74-h-old biofilms. Experiment group 4 was exposed six times per day (at 8, 10, 12 a.m., 2, 4, 6 p.m.) with CS, a total of fifteen times in 74-h-old biofilms. Each exposure time was 5 minutes (simulate the real smoking time of smokers). The culture medium was changed twice daily (8 a.m. and 6 p.m.) (Oral sugar levels rise after 8 a.m. for breakfast and 6 p.m. for dinner). The hydroxyapatite disks were washed with distilled water three times a day (8 a.m., 1 p.m., 6 p.m.) (Simulate cleaning mouth after breakfast, lunch, and dinner) for the control group and all CS exposure groups. This study is approved by the ethics committee/institutional review board of the Department of Preventive Dentistry, School of Dentistry, Institute of Oral Bioscience, Jeonbuk National University. All experimental protocols were approved by the Department of Preventive Dentistry, School of Dentistry, Institute of Oral Bioscience, Jeonbuk National University. The author confirms that all methods were carried out by relevant guidelines and regulations and informed consent had been obtained from the subject.

**Fig 2:**
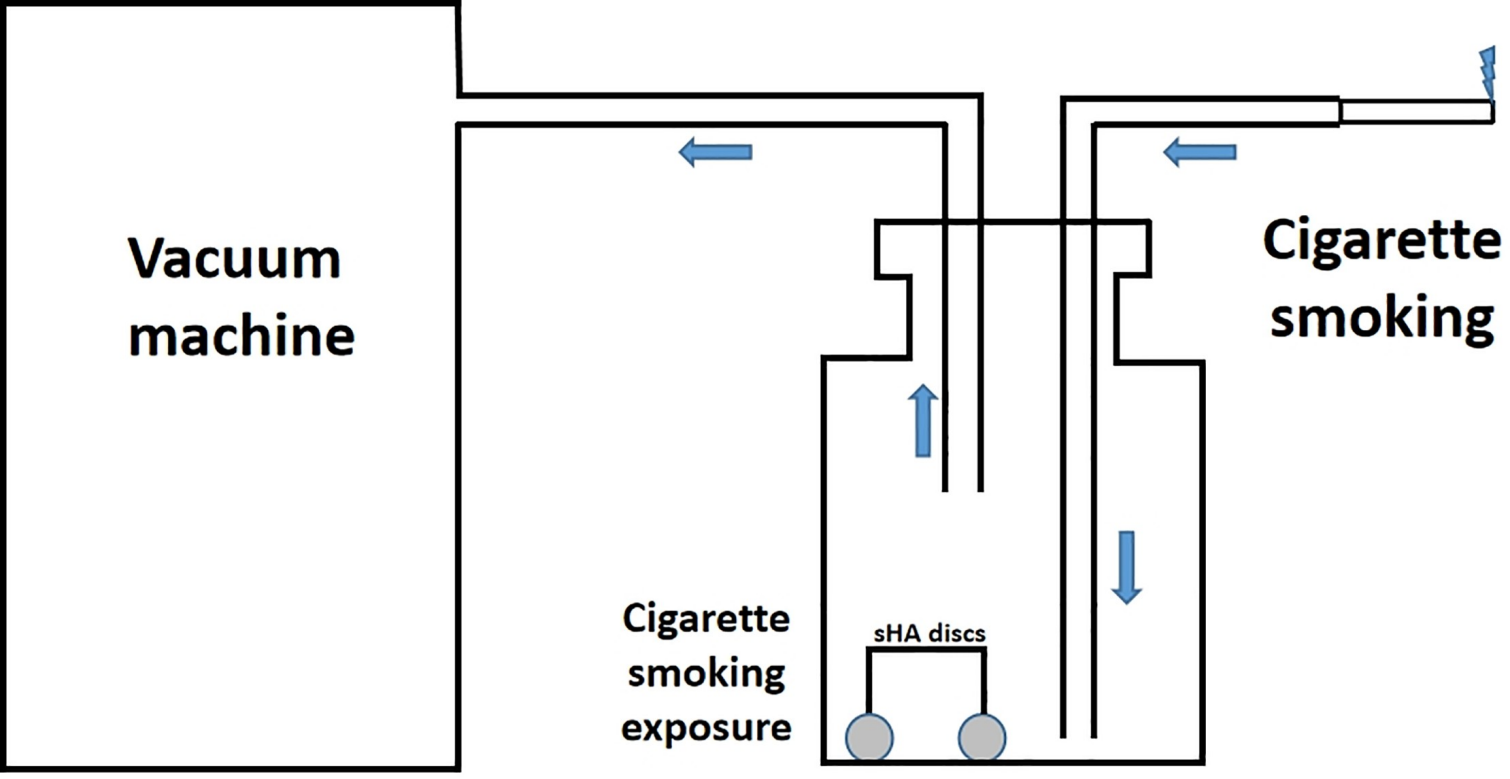
The smoking device.

The microenvironment of plaque is easily affected by various bacteria and external factors. To minimize the influence of external factors and improve the internal validity of the results, and *in vitro* method was considered. Considering that *S*. *mutans* is a facultative anaerobe, a glass container was designed to allow slight air to enter while CS was inhaled to avoid the effect of complete hypoxia on the growth of *S*. *mutans* biofilm. At the time of exposure, the saliva-coated hydroxyapatite discs were taken out from the culture medium, placed in a sterile glass container, and a cigarette was taken using a vacuum machine to simulate cigarette gas in the mouth during smoking, each exposure time was 5 minutes. After exposure, saliva-coated hydroxyapatite discs were returned to the culture medium. This device simulates CS flow has been verified in a previous study (Fig 2) [14].

In this experiment, a popular cigarette in Korean supermarkets was selected. Marlboro (tar: 8.0 mg; nicotine: 0.7 mg), has the highest tar and nicotine content per cigarette of all cigarette brands. We used vacuum machines to provide smoking force.

### Microbiological and biochemical biofilm analyses

The dry weight and CFUs in the homogenized suspension were analyzed. Briefly, the 74-h-old biofilms on the saliva-coated hydroxyapatite disc were transferred into 2 ml of 0.89% NaCl and sonicated in an ultrasonic bath for 10 min to disperse the biofilms. The dispersed solution was re-sonicated at 7W for 30 s after adding 3 ml of 0.89% NaCl (VCX 130PB; Sonics and Materials, Inc., Newtown, CT, USA). For the determination of CFUs count, an aliquot (0.1 ml) of the homogenized solution (5 ml) was serially diluted, plated onto brain heart infusion (BHI; Difco, Detroit, MI, USA) agar plates, and then incubated under aerobic conditions at 37°C to determine the CFUs count [15, 16].

For the determination of the dry weight and amount of water-insoluble/soluble EPSs and IPSs, the remaining solution (4.9 ml) was centrifuged (3000 ×g) for 20 min at 4°C. The biofilm pellet was resuspended and washed twice in the same volume of water. Mix the water-washed the biofilms pellet with 95% alcohol and put it in a refrigerator at -20°C for at least 18 h to precipitate the water-soluble extracellular polysaccharides (EPSs). Then calculate the content of water-soluble EPSs in the biofilms. The washed biofilms pellet was evenly divided into two portions, lyophilized, and weighed to determine the dry weight. One part used 1 N sodium hydroxide to extract water-insoluble EPSs from the dried precipitate. The other part was used to calculate the content of intracellular polysaccharides (IPSs), as detailed elsewhere [17].

The final pH values of the old culture media were also determined during the experimental period using a glass electrode (Beckman Coulter Inc., Brea, CA, USA) to investigate the change in acidogenicity of *S*. *mutans* biofilms by the CS exposure.

### CLSM analysis

#### Live and dead bacterial cells staining

CLSM analysis was performed to confirm the results of microbiological and biochemical studies. To investigate the difference in bacterial cells, the 74-h-old biofilms were stained at room temperature in the dark for 30 min using the Film Tracer LIVE/DEAD Biofilm viability kit L10316 (Invitrogen, Molecular Probes Inc., Eugene, OR, USA). The final concentrations of SYTO®9 and propidium iodide (PI) were 6.0 and 30 μM, respectively. This viability kit was based on plasma membrane integrity to determine live and dead cells. In this study, we regarded the cells with intact membranes (green) as live cells, whereas cells with damaged membranes (red) were regarded as dead cells. The excitation/emission wavelengths were 480/500nm for SYTO®9 and 490/635nm for PI for collecting the fluorescence. The stained live and dead bacterial cells were observed with an LSM 510 META microscope (Carl Zeiss, Jena, Germany) equipped with argon-ion and helium–neon lasers. All confocal fluorescence images were taken with an EC Plan-Neofuar 10x/0.30 M27 objective lens. A stack of slices in 6.4 μm step sizes was captured from the top to the bottom of the biofilms. The biovolume and thickness of live and dead cells were quantified from the entire stack using COMSTAT image-processing software. The biovolume is defined as the volume of the biomass (μm^3^) divided by the substratum (hydroxyapatite surface) area (μm^2^). The three-dimensional architecture of the biofilms was visualized using ZEN 2.3 (blue edition) (Carl Zeiss Microscopy GmbH, Jena, Germany). The original confocal data was uploaded to ZEN 2.3 software and the intensity of green and red fluorescence in the full thickness of biofilms layers were captured automatically. The software reconstructed the 2-dimensional intensity of fluorescence in all the layers to a 3-dimensional volume stack [18].

#### EPS staining

The EPSs of 74-h-old biofilms were also investigated by simultaneous in situ labeling as described elsewhere [19]. Briefly, Alexa Fluor® 647-labeled dextran conjugate (1 μM, 10,000 MW; absorbance/fluorescence emission maxima 647/668 nm; Molecular Probes Inc., Eugene, OR, USA) was added to the culture medium during the formation of *S*. *mutans* biofilms (at 0, 21, 31, 45, 55, 69 h) to label the newly formed EPSs. As described above, the stained EPSs were observed with an LSM 510 META microscope (Carl Zeiss, Jena, Germany) (objective: EC Plan Neofuar 10x/0.30 M27) equipped with argon-ion and helium-neon lasers and visualized using ZEN 2.3. A stack of slices in 7.8 μm step sizes was captured from the top to the bottom of the biofilms. Four independent experiments were performed, and five image stacks per experiment were collected. The EPSs biovolume and thickness were quantified from the confocal stacks using COMSTAT.

### Biofilm density

The density of the 74-h-old *S*. *mutans* biofilms were calculated using the dry weight, which was derived from the biochemical study above, and the total biovolume of the biofilms (live cells + dead cells + EPSs), which was derived from the CLSM study above. The biofilm density (μg/μm^3^) is defined as the dry weight (μg/μm^2^) divided by the total biovolume of the biofilms (μm^3^/μm^2^) [20].

### Statistical analysis

All experiments (except CLSM and SEM) were performed in duplicate, and at least six different experiments were conducted. The data are presented as mean ± standard deviation. Inter-group differences were estimated using a one-way analysis of variance, followed by a post hoc multiple comparisons (Tukey) test to compare multiple means (IBM SPSS Statistics for Windows, version 17) (IBM Corp., Armonk, n.y., USA). Values were considered statistically significant when the *p*-value was < 0.05.

## Result

### The difference in *S*. *mutans* biofilms formation

**Fig 3:**
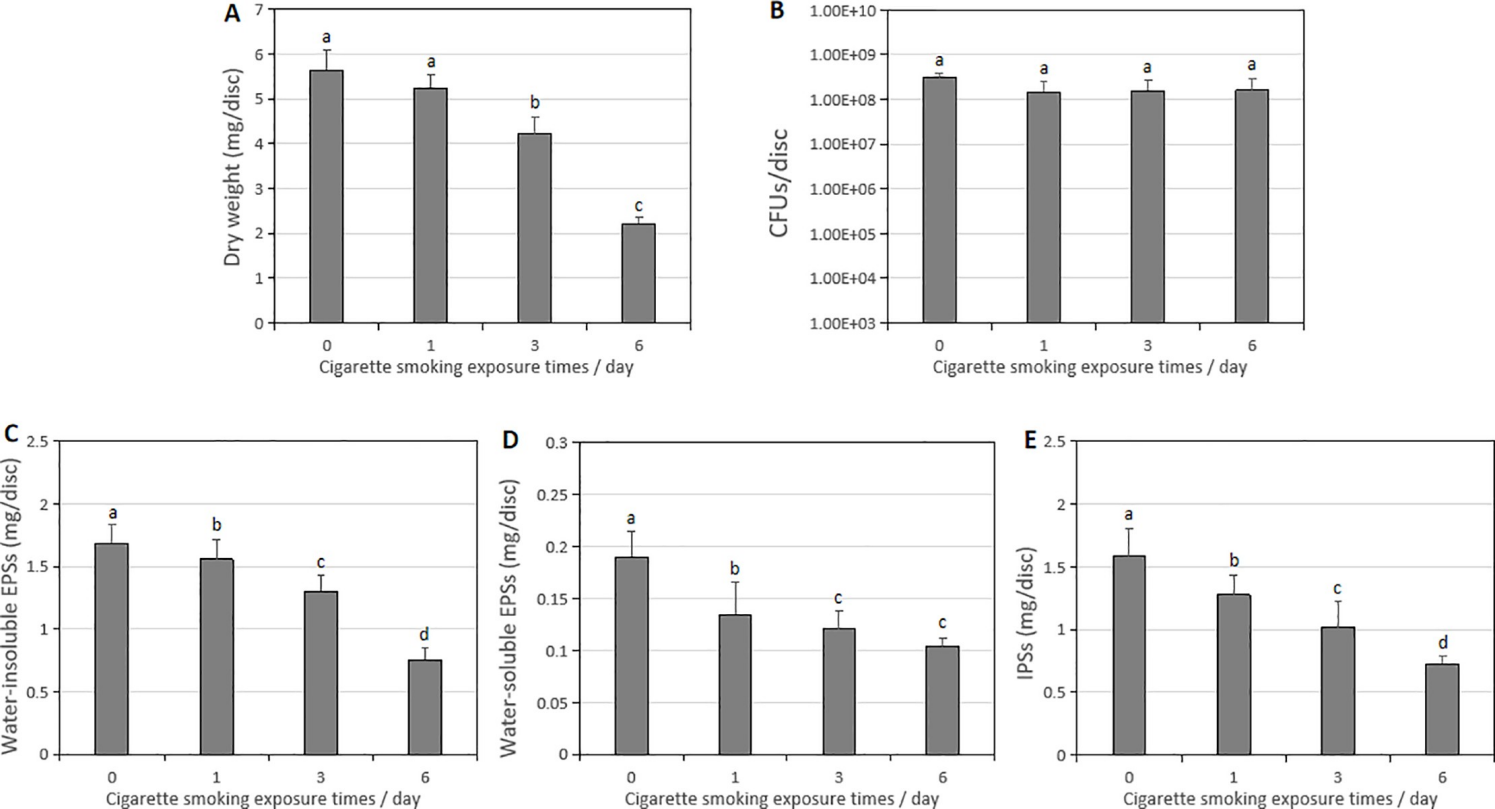
Change in the microbiological and biochemical composition of the 74-h-old *S*. *mutans* UA159 biofilms exposure with CS. (**A**) Dry weight. (**B**) CFUs. (**C**) Water-insoluble EPSs. (**D**) Water-soluble EPSs. (**E**) IPSs. Data represent mean ± standard deviation. Fig 3A and 3C and 3D and 3E, *p* < 0.05. Fig 3B, *p* > 0.05 *p < 0.05: significantly different from each other. *p* > 0.05: values followed by the same superscript are not significantly different from each other.

74-h-old *S*. *mutans* biofilms formation was inhibited by CS exposure. In the experimental results of microbial and biochemical studies, all CS exposure groups reduced the dry weight accumulation, water-insoluble/soluble EPSs, and IPSs formation of the 74-h-old *S*. *mutans* biofilms in a frequency-dependent manner, and the lowest values in the six times/day CS exposure group compared with the control group and other CS exposure groups (Fig 3A–3E, *p* < 0.05). There was no significant difference in bacterial activity (CFUs) counts among all CS exposure groups (Fig 3B; *p* > 0.05).

### The pH value in the culture medium

**Fig 4:**
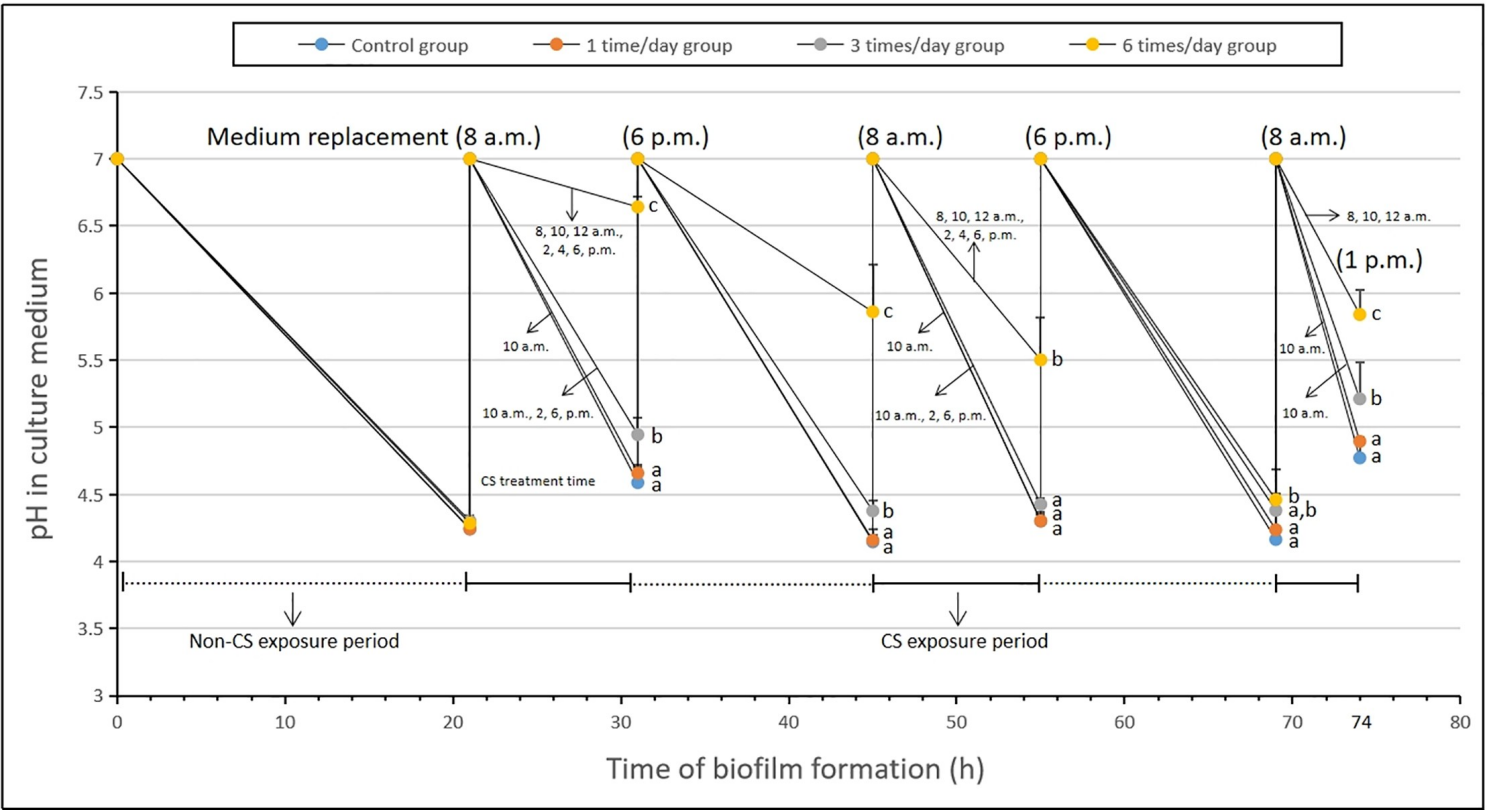
Change in the pH values of old culture medium exposure with CS during 74-h-old *S*. *mutans* UA159 biofilms formation. Data represent mean ± standard deviation. Values followed by the same superscript are not significantly different from each other (*p* > 0.05).

Fig 4 shows the pH change in the culture medium by different times of CS exposure during 74-h-old *S*. *mutans* biofilms formation. Acidogenicity during *S*. *mutans* biofilms formation was affected by CS exposure. Although the acidogenicity of *S*. *mutans* biofilms before the CS exposure was not different from each other (*p* > 0.05) (0 ~ 21 h of biofilm formation). But the pH in the culture medium started to change after exposure with CS, the acidogenicity was inhibited at all of the CS exposure groups (*p* < 0.05) (21 ~ 74 h). The inhibition of acidogenicity of biofilms was enhanced with the increase of CS exposure frequency, and the highest inhibition ability was shown in the six times/day CS exposure group (*p* < 0.05). Furthermore, in all of the CS exposure periods and non-CS exposure periods (21 ~ 74 h), the pH values of the culture medium of *S*. *mutans* biofilms in the six times/day CS exposure group were significantly different from the control group (*p* < 0.05).

### Bacterial biovolume and thickness

**Fig 5:**
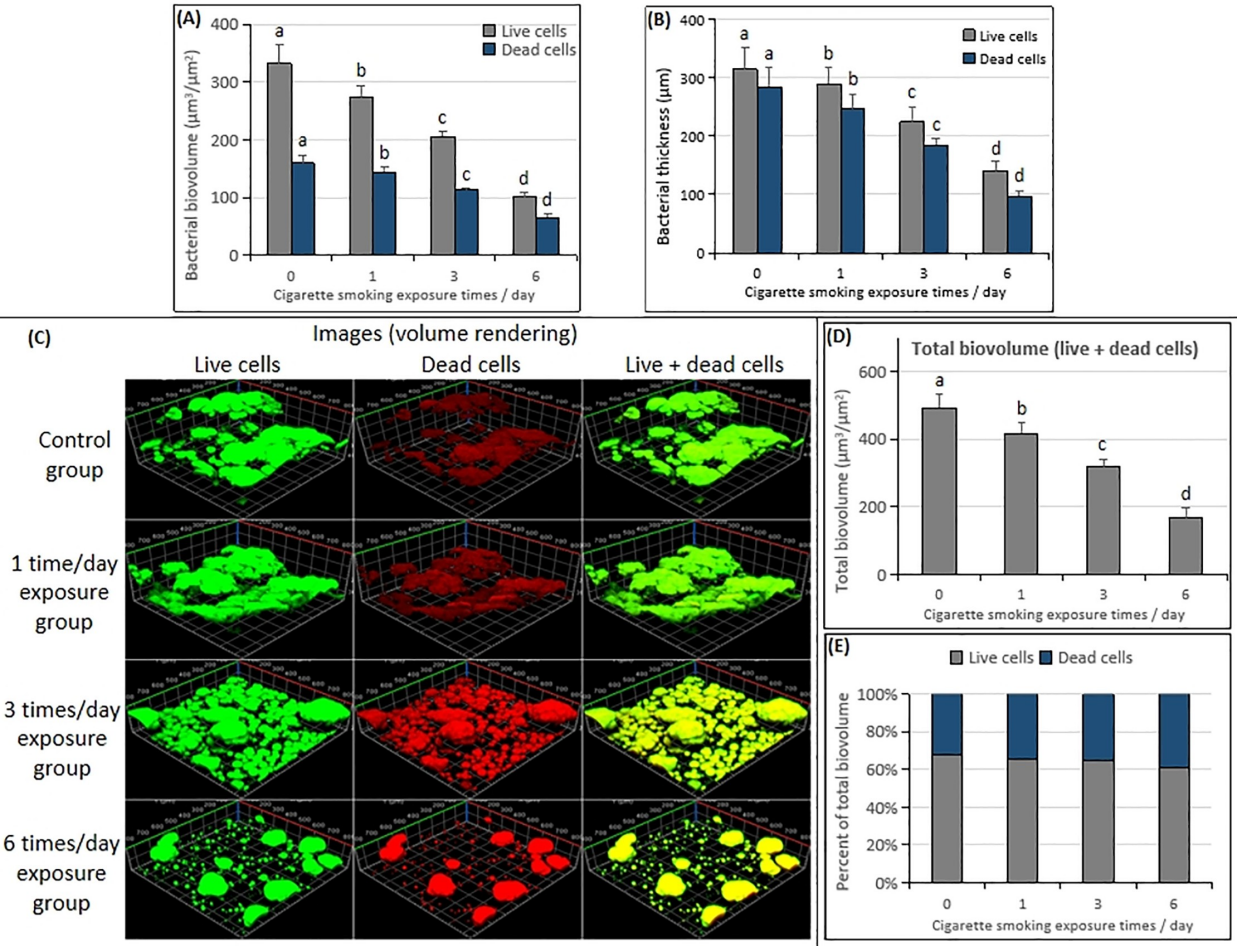
Change in CLSM of bacterial cells in the 74-h-old *S*. *mutans* UA159 biofilms exposure with CS. (**A**) Bacterial biovolume. (**B**) Bacterial thickness. (**C**) Representative confocal images. (**D**) Total biovolume (live + dead cells). (**E**) Percent of the total biovolume. Significantly different from each other (*p* < 0.05).

To further evaluate the effect of CS on biofilm components and structure, the CLSM analysis was performed. The bacterial biovolume and thickness of live or dead cells of the 74-h-old *S*. *mutans* biofilms showed different results under different times of CS exposure (Fig 5). The lowest values of bacterial biovolume and thickness of live or dead cells and total biovolume of the were detected in the six times/day CS exposure group compared with the other CS exposure groups and control group in the 74-h-old *S*. *mutans* biofilms (Fig 5A and 5B and 5D; *p* < 0.05). In the percent of total biovolume, the proportion of live cell biovolume in each exposure group was between 60% and 70% (Fig 5E). The three-dimensional image of bacterial microcolonies of 74-h-old *S*. *mutans* biofilms showed that with the increased frequency of CS exposure, the morphology was changed into a lower number of the live/dead cells microcolonies with a smaller size and a loose arrangement (Fig 5C).

### EPS biovolume and thickness

**Fig 6:**
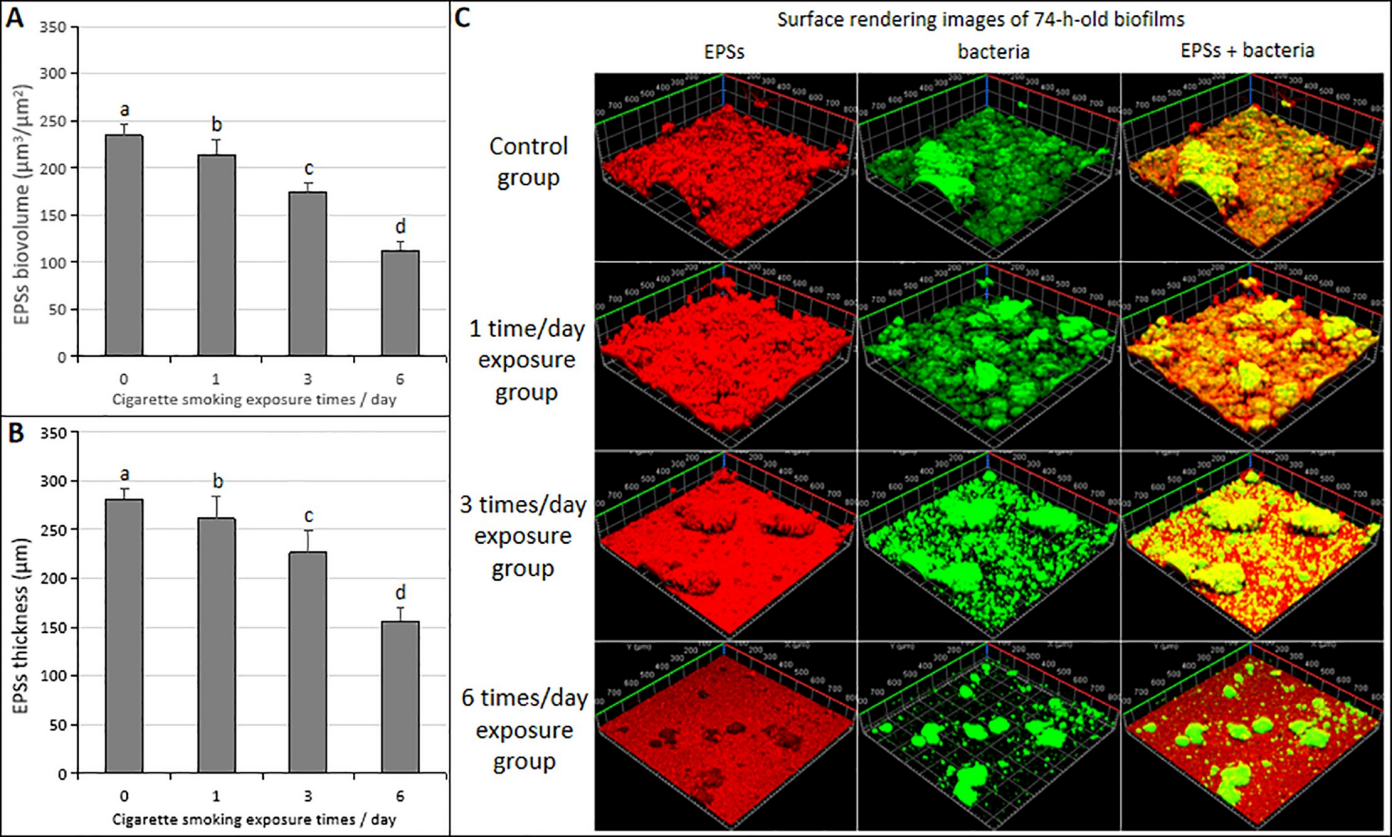
Change in CLSM of EPSs in the 74-h-old *S*. *mutans* UA159 biofilms exposure with CS. (**A**) EPSs biovolume. (**B**) EPSs thickness. (**C**) Representative confocal images. Significantly different from each other (*p* < 0.05).

The biovolume and thickness of EPSs in the 74-h-old *S*. *mutans* biofilms are influenced by the different times of CS exposure. The mean biovolume and thickness of EPSs of the 74-h-old *S*. *mutans* biofilms in the six times/day CS exposure group showed the lowest values compared with the other CS exposure groups and control group (Fig 6A and 6B; *p* < 0.05). With the increase of CS exposure frequency, the anti-EPSs formation increased in a frequency-dependent manner. EPSs and bacterial microcolony images of 74-h-old *S*. *mutans* biofilms showed minimal concentrations of homogeneous structures of EPSs covering and surrounding bacterial microcolonies in the six times/day CS exposure group (Fig 6C).

### The difference in biofilm density

**Fig 7:**
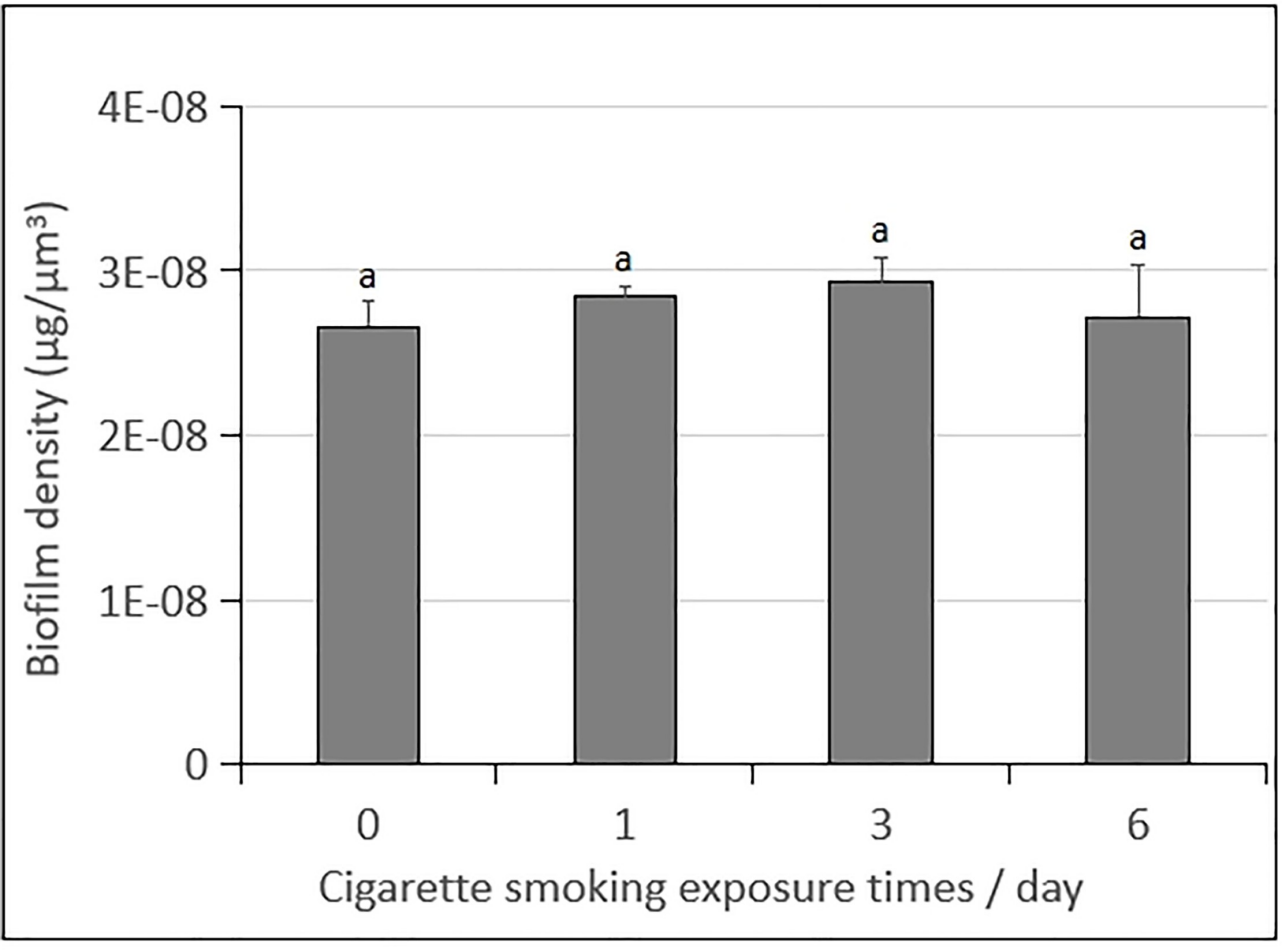
Change in the biofilm density of 74-h-old *S*. *mutans* UA159 biofilms exposure with CS. Values followed by the same superscripts are not significantly different from each other (*p* > 0.05).

The density of *S*. *mutan*s biofilms were calculated to investigate the difference in biofilm compactness according to different times of CS exposure. As shown in Fig 7, there was no significant difference in biofilm density in all the CS exposure groups and the control group (*p* > 0.05).

**Fig 8:**
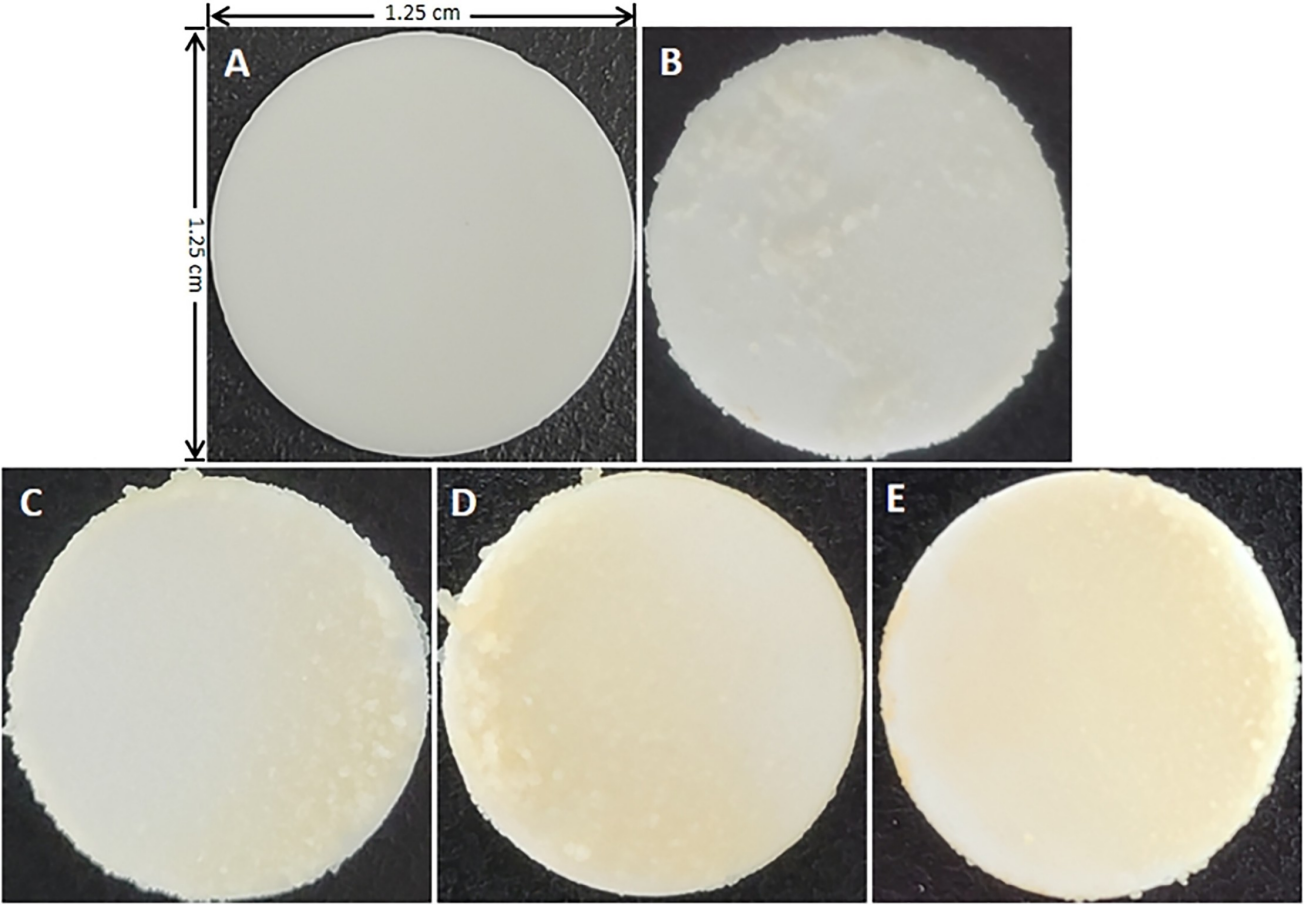
Surface morphology of hydroxyapatite disks before the experiment and each group after 74-h-old incubation. (**A**) Before the experiment. (**B**) Control group. (**C**) 1 time/day CS exposure group. (**D**) 3 times/day CS exposure group. (**E**) 6 times/day CS exposure group.

Fig 8 showed the surface morphology of each group of hydroxyapatite disks after 74-h-old incubation. The surface of the hydroxyapatite disk in the six times/day CS exposure group showed the largest area of the yellow film (a mixture of chemical substances produced by cigarette combustion) covering compared to the other CS exposure groups and the control group. And the accumulation of biofilm on the surface of the yellow film covering the area was less than that on the surface of white hydroxyapatite.

## Discussion

This study was to investigate the differences in growth, virulence (EPSs and acidogenicity), and viability of cariogenic biofilms according to the different times of CS exposure (simulate different smoking times each day) using an *S*. *mutans* biofilm model *in vitro*. *S*. *mutans* biofilm model is unable to precisely mimic the complex microbial community in dental biofilms, the mono-species biofilm is advantageous in examining the mechanisms of *S*. *mutans* in biofilms, in biofilms with sufficient performance to validate the data reproducibility and reduce variance [17, 21, 22].

As shown in Fig 3A, all CS exposure groups reduce the dry weight accumulation of 74-h-old *S*. *mutans* biofilm. The six times/day CS exposure group showed the lowest dry weight accumulation compared with the control group and other CS exposure groups (Fig 3A; *p* < 0.05). CS inhibited the dry weight accumulation of *S*. *mutans* biofilm in a frequency-dependent manner. There was no significant difference in the bacterial activity between all CS exposure groups and the control group (Fig 3B; *p* > 0.05). CLSM revealed that the live/dead cells bacterial biovolume, bacterial thickness, and total biovolume in 74-h-old *S*. *mutans* biofilms were frequency-dependently reduced by CS exposure (Fig 5A and 5B and 5D; *p* < 0.05). The lowest values were shown in the six times/day CS exposure group. With the increase of CS exposure times, the number of live/dead cell microcolonies decreased and the arrangement of microcolonies was loose. However, the morphology of microcolonies did not change significantly, microcolonies in each treatment group that were similar to those in the control group could be observed (Fig 5C). In addition, according to the results of the bacterial activity (Fig 3B), the live/dead cell microcolonies (Fig 5C), and the proportion of live cell biovolume (Fig 5E), the CS exposure had no bactericidal or killing effect on the growth of *S*. *mutans* biofilms.

Polysaccharides account for up to 40% of the dry weight of dental biofilms is, which are mostly synthesized by microbial glucosyltransferases [23]. Therefore, the reduction of biofilm biomass is directly related to the reduction of polysaccharides in the whole biofilm matrix. The complex structural integrity of a biofilm is mainly determined by the density of the formation, volume, structural integrity, and stability of which is also associated with EPSs [6, 24]. In this study, the effects of CS on the synthesis of water-soluble/insoluble EPSs and IPSs during the formation of *S*. *mutans* biofilms were investigated. All the CS exposure groups in the 74-h-old *S*. *mutans* biofilms decreased the synthesis of water-soluble/insoluble EPSs and IPSs, the lowest values were detected in the six times/day CS exposure group compared to the control group and other CS exposure groups (Fig 3C–3E; *p* < 0.05). CS exposure inhibited the synthesis of polysaccharides of 74-h-old *S*. *mutans* biofilm in a frequency-dependent manner, which was consistent with the CLSM findings of the biovolume and thickness of EPSs (Fig 6; *p* < 0.05). Therefore, CS had an obvious damage effect on the EPSs formation of biofilms, and the decreased biovolume and thickness of EPSs may affect the growth height of bacterial cells (Fig 6).

Acid production by carbohydrate and acid tolerance in a low pH environment is one of the main toxicity characteristics of *S*. *mutans* [6], which are closely associated with enamel demineralization and the formation of dental caries [25–27]. In this study, acid production of *S*. *mutans* biofilms was found to be inhibited by all CS exposure groups through analyzing pH values in the old culture medium, and the highest inhibitory rate was detected in the six times/day CS exposure group (Fig 4; *p* > 0.05).

The above results indicated that the CS exposure inhibited the growth of *S*. *mutans* biofilms in a frequency-dependent manner *in vitro*, which was well supported by the measurements of biofilm dry weight (Fig 3A), EPSs (Figs 3C and 3D and 6), IPSs (Fig 3E), acidogenic (Fig 4), bacterial count (live or dead cells) (Fig 5A) and total biovolume (Fig 5D). The six times/day CS exposure group showed the highest inhibition compared to the control group, and other CS exposure groups. No significant differences in the bacterial activity (CFUs counts; Fig 3B) and biofilm density (Fig 7) were detected between all CS exposure groups and the control group. Meanwhile, CLSM revealed a similar ratio of live cells and dead cells in the total biovolume between all CS exposure groups and the control group (Fig 5E). These results suggested that CS did not inhibit the growth of *S*. *mutans* biofilms by sterilizing or killing cells.

The author further analyzed the mechanism of CS exposure in inhibiting the growth of *S*. *mutans* biofilm *in vitro*. It is considered that CS exposure altered the colonization environment on the surface of the hydroxyapatite disc where *S*. *mutans* biofilms were attached. The hydroxyapatite disks in all CS treatment groups at 74 h incubation showed that the surface of the hydroxyapatite disks was covered with a yellow film by the mixture of chemical substances produced by cigarette combustion attached to the surface of the hydroxyapatite disks (Fig 8). Cigarette combustion produces more than five thousand chemicals that contain various harmful substances [28–30]. The area of the yellow film on the surface of the hydroxyapatite disk was significantly larger in the six times/day exposure group than that of other CS exposure groups and the control group. Compared with the normal white hydroxyapatite disk in the control group, the accumulation of biofilms on the yellow film surface in the six times/day exposure group was less than that on the normal hydroxyapatite disk surface, and the formation of *S*. *mutans* biofilms could not even be seen on some areas of the yellow film surface (Fig 8). This may be since the mixture of chemical substances produced by cigarette combustion attached to the surface of the hydroxyapatite disc, altered the environment of the initial hydroxyapatite disc that blocked the colonization of *S*. *mutans* biofilms on the surface of the chemical mixture. This study showed that with the increased frequency of CS exposure, the novel mixture of chemical substances produced by cigarette combustion continuously covered the surface of *S*. *mutans* biofilms, inhibited the growth and the new colonization of *S*. *mutans* biofilm on the surface of the chemical mixture. In the hydroxyapatite disks of the CS exposure groups, with the increased frequency of CS exposures, the available area for the colonization and growth of *S*. *mutans* biofilms became smaller and smaller. As a result, the lowest biofilm dry weight, EPSs, IPSs, acidogenic bacterial count (live or dead cells), and total biovolume were detected in the six times/day CS exposure group. CLSM revealed that the thickness of bacteria (live or dead cells) was negatively correlated with the frequency of CS exposures, The lowest bacterial thickness detected in the six times/day CS exposure group also confirmed that the coverage of mixture chemical substances produced by cigarette combustion continuously covered on the surface of the *S*. *mutans* biofilms inhibited the increase in bacterial thickness (Fig 5B). Mixture chemical substances produced by cigarette combustion that were attached to the surface of the hydroxyapatite disk also resulted in the color change of the disk. In practice, the mixture of chemical substances of CS attached to the surface of the teeth caused the color change [31].

The results of this study support CS exposure in inhibiting *S*. *mutans* biofilms growth was inconsistent with previous ones that smoking is closely related to the high incidence of dental caries [7–13], which could be explained by this study is *in vitro* and lacked the influence of the oral immune system on the growth process of cariogenic biofilm. One study demonstrated that salivary secretory IgA (s-IgA), pH, and flow rates were of great significance in oral mucosal immunity, and salivary S-IgA antibodies generated by the mucosal immune system were an important role in the immune response against dental caries. Low concentrations of salivary sIgA were correlated with a higher prevalence of dental caries in smokers [32]. It has been shown that CS impairs saliva function, which has an important role in promoting caries. In addition, both plasma and saliva activities of lactic dehydrogenase (LDH), aspartate aminotransferase (AST), and amylase are significantly reduced by external addition of aldehydes or CS exposure [33]. A decreased buffering effect of the saliva of smokers, and higher numbers of *Lactobacilli* and *S*. *mutans* increase the susceptibility to dental caries [34], which may explain the relationship between CS and the high incidence of dental caries.

In general, the results *in vitro* study supported the fact that CS exposure inhibited the growth of *S*. *mutans* biofilms because the mixture of chemical substances produced by cigarette combustion changed the colonizing environment on the surface of the hydroxyapatite disc, rather than sterilizing or killing cells. In this study, the frequency of CS exposure was the main variable, and there was no interference from other oral factors. Although only one cariogenic bacterium was included in this study, these findings provide a novel insight into clarifying the biological effects of CS on cariogenic biofilm growth.

## Conclusion

CS exposure inhibited the growth of *S*. *mutans* biofilm *in vitro* study, the anti-cariogenic biofilm formation was enhanced with a dose (frequency)-dependent at which frequency has more influence in the present findings.
